# Verdinexor Targeting of CRM1 is a Promising Therapeutic Approach against RSV and Influenza Viruses

**DOI:** 10.3390/v10010048

**Published:** 2018-01-21

**Authors:** Jennifer A. Pickens, Ralph A. Tripp

**Affiliations:** Department of Infectious Diseases, University of Georgia, Athens, GA 30602, USA; jenniferapickens@gmail.com

**Keywords:** Chromosome Region Maintenance 1, Influenza, Respiratory syncytial virus, Verdinexor, KPT-335, antiviral

## Abstract

Two primary causes of respiratory tract infections are respiratory syncytial virus (RSV) and influenza viruses, both of which remain major public health concerns. There are a limited number of antiviral drugs available for the treatment of RSV and influenza, each having limited effectiveness and each driving selective pressure for the emergence of drug-resistant viruses. Novel broad-spectrum antivirals are needed to circumvent problems with current disease intervention strategies, while improving the cytokine-induced immunopathology associated with RSV and influenza infections. In this review, we examine the use of Verdinexor (KPT-335, a novel orally bioavailable drug that functions as a selective inhibitor of nuclear export, SINE), as an antiviral with multifaceted therapeutic potential. KPT-335 works to (1) block CRM1 (i.e., Chromosome Region Maintenance 1; exportin 1 or XPO1) mediated export of viral proteins critical for RSV and influenza pathogenesis; and (2) repress nuclear factor κB (NF-κB) activation, thus reducing cytokine production and eliminating virus-associated immunopathology. The repurposing of SINE compounds as antivirals shows promise not only against RSV and influenza virus but also against other viruses that exploit the nucleus as part of their viral life cycle.

## 1. Introduction

Influenza A virus (IAV) and respiratory syncytial virus (RSV) are two leading causes of viral respiratory tract illnesses, resulting in considerable mortality and morbidity. The very young, elderly, and immunocompromised are at risk for developing disease associated with IAV or RSV infections. Both viruses share similar peak seasons that begin in the fall and proceed into early spring. According to the World Health Organization, the IAV and influenza B viruses (IBV) are responsible for seasonal epidemics causing approximately 3–5 million cases and upwards of 500,000 deaths worldwide [1]. The annual number of cases and hospitalizations vary depending on epidemic or pandemic years.

Current circulating influenza viruses consist of influenza A H1N1 and H3N2 strains, as well as influenza B Victoria-like and Yamagata-like strains [1]. The influenza virus is typically self-limiting, resulting in generally mild upper respiratory illness; however, in patients with underlying medical conditions, complications of infection can lead to severe disease and fatalities. Pneumonia, bronchitis, otitis media, and sinusitis are the common complications following IAV infections [2,3,4]. Like the influenza virus, RSV is an agent linked to viral respiratory illnesses, particularly in the very young and elderly [2,5,6,7,8]. RSV infections typically result in mild upper respiratory tract illness, but in severe cases, can cause bronchiolitis and pneumonia requiring hospitalization [8]. Annually, there are approximately 1 million pediatric cases in the US, where 70% of infants are infected by the first year of life and nearly every child by the age of 3 [2,6]. Prior exposure to RSV does not induce lifelong durable immunity; thus, the elderly (>65 years of age) are at high-risk for serious and potentially life-threating complications associated with infection [5,9]. Each year there are nearly 10,000 deaths and over 177,000 hospitalizations reported for adults over the age of 65 in the US due to RSV [10].

Antiviral therapeutics generally target viral proteins needed for replication and many have proven to be ineffective at preventing influenza- and RSV-associated diseases due to features associated with the emergence of drug resistance. To avoid these issues, research efforts have focused on identifying and evaluating new antiviral drugs that target cellular factors and pathways hijacked by the virus used in replication, rather than focus on a viral component that may readily change under the selective pressure of the drug and/or immune system. The nuclear trafficking of viral components is a critical stage in the influenza virus and RSV replication. Inhibition of nuclear export not only attenuates viral replication but also impacts the regulation of immune-modulating factors [11,12,13,14]. In this review, we summarize the current understanding of targeting the host CRM1 (Chromosome Region Maintenance 1; exportin 1 or XPO1) dependent export pathway as a broad-spectrum antiviral therapeutic strategy against the influenza virus and RSV.

## 2. Trafficking of Influenza and RSV

### 2.1. Influenza

Influenza viruses are enveloped viruses of the *Orthomyxoviridae* family, comprised of a segmented, negative-sense single stranded RNA genome. The viral ribonucleoprotein (vRNP) complex consists of each viral RNA segment encapsulated by nucleoproteins (NP) and carrying its own heterodimeric RNA dependent RNA polymerase, comprised of polymerase basic 1 (PB1) polymerase basic 2 (PB2) and polymerase acidic (PA) subunits [15,16,17,18]. Each influenza subtype is defined by antigenically distinct hemagglutinin (HA) and neuraminidase (NA) glycoproteins (e.g., H1N1) with 18 HA and 11 NA subtypes identified in reservoir species such as shore birds, poultry and swine [19,20,21]. In humans, IAV infections are typically isolated to the upper respiratory tract. Upon infection, the HA facilitates viral entry by binding to α2, 6 linked-sialic acids (*N*-acetylneuraminic acid) of the surface of respiratory epithelium [22,23]. Proteolytic cleavage of the HA0 to the HA1 and HA2 subunits is required for viral infection. The M2 proton channel acidification of the endosome results in a conformational change in the HA, liberating the HA2 fusion peptide and thereby mediating fusion of the viral envelop with the host endosomal membrane and release of the vRNP complex into the cytoplasm [24,25,26,27,28,29]. The vRNPs are trafficked to the nucleus and subsequently imported by a group of proteins involved in transporting molecules between the cytoplasm and the nucleus of a eukaryotic cell called the karyopherin proteins that include importin-α [18,30,31]. Although all of the protein subunits of the vRNP complex (NP, PA, PB1 and PB2) encode nuclear localization signals (NLS), it is the NP subunit that has been shown to be sufficient at modulating nuclear import of vRNPs, encoding 3 NLS motifs [17,18,32,33]. Once in the nuclear compartment, the viral RNA (vRNA) undergoes replication and transcription, generating progeny vRNPs that are then transported out of the nucleus and trafficked to the host membrane for assembly into progeny virions. IAV use the CRM1 nuclear export pathway to facilitate the nuclear to cytoplasmic transport of proteins bearing nuclear export signals (NES) [13,34]. Three viral proteins have been shown to be important for nuclear export. Although NP is the only protein of the vRNP complex to have a known NES, the viral M1 and nuclear export protein (NEP) have also been shown to be critical for vRNP complex nuclear export, where infected cells that lack M1 inhibit vRNP complex nuclear export (for review, see [13,34,35,36]).

### 2.2. RSV

Belonging to the *Pneumoviridae* family, RSV is an enveloped virus containing a non-segmented, negative-sense single stranded RNA genome [37]. The packaging of the RSV RNA into vRNPs is through encapsulation by nucleocapsid (N) protein. The complex contains the viral RNA dependent RNA polymerase, compromised of the large polymerase (L) bound to its cofactors, the phosphoprotein (P) and the transcription elongation factor (M2-1) [38,39]. Within the virion, the vRNP is associated with the RSV matrix protein (M) by means of M2-1 protein. The RSV fusion (F) protein and attachment glycoprotein (G) mediate viral entry in airway epithelial cells [40,41,42,43,44]. RSV gains entry through G protein binding of glycosaminoglycans (GAG: heparin sulfate and chondrion sulfate GAG), followed by primary binding of RSV F to nucleolin and the embedding of cholesterol-rich micro domains at primary cilium and microvilli at the apical surface of respiratory epithelium [42,44,45,46,47]. Upon RSV F-mediated fusion of the viral envelop with the host plasma membrane, the vRNP dissociates from the M protein, delivering the viral genome to the cytoplasm allowing for RSV replication to occur. The replication process is not solely localized to the cytoplasm; although the RSV matrix (M) protein is trafficked to the nucleus early in the viral life cycle, at later time points it is found associated with vRNPs within replication centers called inclusion bodies [38,48,49]. The RSV M protein contains both NLS and NES motifs, shuttling into the nucleus via importin-α and out through CRM1 [12,50]. It is unclear what role the M protein plays within the nuclear compartment during early infection, but it may be linked to reductions in host transcription levels in RSV-infected cells [48]. The subcellular localization of RSV M protein within the nucleus is a critical step in the infection of cells, where CRM1 inhibition with the irreversible inhibitor leptomycin B (LMB) results in M protein nuclear accumulation and reduced RSV replication [12]. 

## 3. Disease Interventions

### 3.1. Influenza Therapeutics and Vaccines

To date, there are two classes of approved anti-influenza virus drugs available that target either the viral M2 or the NA proteins. Amantadine and rimantadine are IAV antivirals, and the mechanism of action of these drugs block the M2 proton channel shortly after viral entry, preventing delivery of the viral genome into the cytoplasm of the infected cell [51]. The M2 inhibitors are active against IAV strains, but not IBV. Widespread drug resistance is common among the amantadines. Resistance has been reported for the pandemic H1N1 (pH1N1) and H3N2 circulating viruses, and the CDC no longer recommends them as an IAV treatment option [52,53,54]. The NA inhibitors, oseltamivir and zanamivir, are the second generation of IAV antiviral drugs that mediate their effect by binding and blocking the enzymatic active site of the viral neuraminidase, thus causing the newly assembled virus to aggregate and preventing viral release and spread of infection to neighboring cells [55,56,57,58,59,60,61]. The NA inhibitors are effective against both IAV and IBV but must be administered within 48 h of infection [61,62,63]. While the rate of drug resistance to the NA inhibitors is not as widespread as with the M2 inhibitors, there have been reports of drug resistance [56,64]. Heightened concerns over the rising number of NA-resistant influenza strains has led to the use of oseltamivir and zanamivir though use is limited to complicated and high-risk IAV cases only [1,56,65]. Additional NA inhibitors have been developed that offer advantages over oseltamivir and zanamivir. Newly licensed Peramivir is now available in the US and utilizes an intravenous administration route, producing the same level of inhibition as compared to oseltamivir (oral) and zanamivir (inhalation) at lower dose concentrations [66,67,68]. Laninamivir, which is currently in phase III clinical trials, demonstrates long-acting inhibition when given as a single daily administration, whereas oseltamivir and zanamivir require twice daily dosing to be effective against influenza [69,70,71,72,73,74].

The influenza vaccine is considered by some as the best defense against influenza infections, but it requires annual administration to be prophylactically effective against seasonal circulating strains [1,65]. The viruses to be included in the influenza vaccine are predicted based on the previous season’s circulating viruses. However, there are often issues with correctly matching the influenza viruses for a particular season, with mismatched seasons showing an increased number of influenza cases as compared to years where the vaccine is well matched to the circulating viruses [65,75]. Even when the vaccine is well-matched, it is still exhibits a range of effectiveness, varying from 10% to as high as 60% with the current 2017 vaccine only demonstrating 10% efficacy against the dominant H3N2 strain [65,75].

Current research efforts are focused on development of a “universal” influenza vaccine and there are several in phase II clinical trials. Ideally, these would protect not only against the strains in circulation but also against emerging novel influenza strains. The premise of these vaccines is to target epitopes (e.g., HA stalk, M1, NP, and PB1 proteins) conserved among the various influenza subtypes and generate a potent neutralizing antibody response [65,75,76,77,78,79,80]. Preliminary data have shown evidence that a “universal” influenza vaccine is feasible, but it is still not clear how efficacious it will be against emerging strains and if it will demonstrate improved efficacy over the available seasonal vaccine [65,76,77,78,79].

### 3.2. RSV Therapeutics and Vaccines

Unfortunately, there is no safe and effective RSV vaccine, despite >50 years of research effort. The choices to control RSV in infants and young children are limited to: (1) palivizumab, a humanized mouse monoclonal antibody against RSV F that is administered prophylactically in high-risk pediatric cases, and (2) ribavirin, a guanosine analogue that exerts an antiviral effect by inhibiting viral RNA synthesis and viral messenger (mRNA) capping [81,82]. The latter is only used to treat severe cases of RSV, despite limited evidence of efficacy and risk of toxicity [81,83,84]. As with other antivirals that target RSV, drug resistance is an issue with ribavirin, where drug resistant mutants limit its use [85,86,87]. Both therapeutics are costly and have only proven to be partially effective against RSV [81,84,88,89,90]. For uncomplicated cases, there are no therapeutic choices other than supportive therapy [9,10].

Despite nearly 50 years of research efforts, no vaccine has been approved for RSV. While there has been progress in the development of a safe and efficacious RSV vaccine, the US Food and Drug Administration (FDA) is hesitant to approve an RSV vaccine, especially after the 1960 failure of the formalin-inactivated alum-precipitated RSV (FI-RSV) vaccine that resulted in enhanced disease, two deaths and hospitalization of 80% of the vaccinated subjects [91,92]. There are a number of candidates in clinical trials that have been reviewed in detail elsewhere [93,94]. Candidate vaccines currently used in clinical trials include live attenuated, subunit, vector and nanoparticle platforms in combination with a variety of different adjuvants. A large portion of the candidate vaccines focus on the RSV F protein, which is critical for viral entry, with the most advanced being the Novavax F-protein virus-like nanoparticle (VLP) vaccine adjuvanted with aluminum hydroxide, which is in phase III for maternal vaccination [95,96]. There is a need for a safe and effective vaccine for those at high risk for development of severe RSV disease (especially preterm infants), and there are only 3 candidate vaccines currently in phase I trials, both are recombinant live attenuated vaccines assessed in 6- to 24-month-old children [97]. These vaccines are attenuated by deletion of critical RSV genes (i.e., M2-2, SH, and NS2), while still eliciting a neutralizing antibody response [97]. Given the tragic history of the FI-RSV vaccine and the public concerns around vaccines, all vaccine candidates are extensively assessed for any counter indications with very few making it to the clinic.

Although ongoing research efforts to develop novel antivirals against RSV have led to several candidates, none have translated into new anti-RSV drugs (Table 1) [88]. Initially, antiviral drug research was aimed towards targeting RSV directly, but drug-induced selective pressures have resulted in mutant RSV that were less sensitive to the drug [98,99]. This illustrates the need for novel anti-RSV drugs that target host-mediated pathways essential for virus replication.

### 3.3. Potential Directions to Discover Novel Therapeutics against RSV and Influenza

Even with the available therapeutics targeting RSV and IAV, these viruses still remain a persistent public health and economic burden. Work is underway to identify novel and efficacious drug alternatives that target every aspect of the viral pathogenesis in the host, as well as block the immune modulating factors shown to cause immunopathology. This approach will lead to the development of novel immunomodulatory drugs, antiviral agents, and vaccines, while also assessing natural products in the treatment of RSV and IAV. Nearly every step within the virus life cycle from viral entry to egress can be targeted, either by interfering with the function of a viral protein itself or through inhibition of the host pathway hijacked by the virus. A brief overview of anti-RSV, anti–IAV and immunomodulatory therapeutics currently under investigation has been provided (Table 1) and recently reviewed elsewhere [93,94,100,101].

## 4. Influenza and RSV-Induced Inflammatory Response

Influenza and RSV are pathogens that predominately infect the respiratory epithelium and must overcome intrinsic barriers to establish infections. Uncomplicated cases of infection by these viruses result in upper respiratory tract illnesses with mild symptoms including sinusitis, cough, sore throat, headache, fever, anorexia and myalgia [1,4,132]. In more complicated cases, the patient may present with severe bronchiolitis, pneumonia, pulmonary function abnormalities, apnea and secondary bacterial infections [1,4,133]. All of these symptoms are linked to a virus-induced inflammatory response. This response is a key defense process designed to protect the host against invading pathogens; however, an uncontrolled and heightened response may be associated with intense tissue damage with the most extreme cases resulting in fatalities [133,134,135,136]. In enhanced IAV disease, pro-inflammatory cytokines and chemokines can cause damage to pulmonary tissues, leading to respiratory dysfunction or acute respiratory distress syndrome (ARDS) [3,133,135,136]. RSV infection has been associated with acute and chronic complications that include recurrent wheezing, allergic airway disease and pulmonary function abnormalities [137,138,139,140,141,142]. Disease severity is greatly associated with an exacerbated innate immune response, where high levels of cytokines and chemokines cause localized tissue pathology and a cytopathic effect.

**Figure 1:**
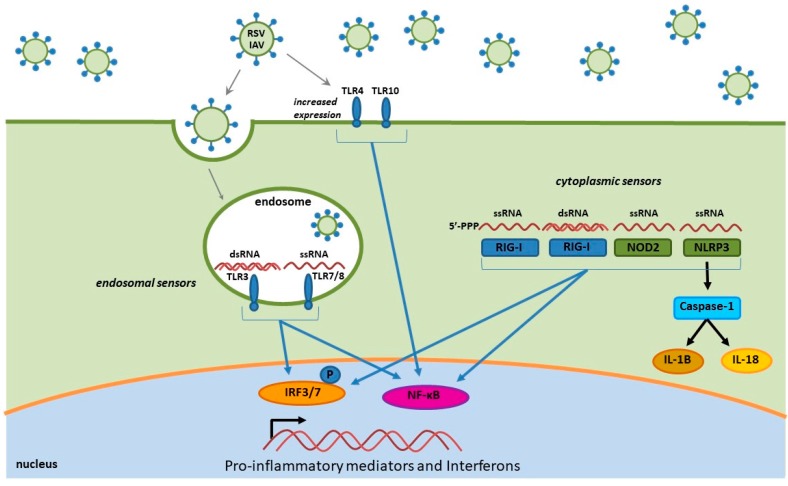
RSV and IAV single-stranded RNA and double-stranded RNA moieties generated during viral replication are detected by both endosomal Toll-like receptors (TLR) 3 and TLR7/8, and cytoplasmic pattern recognition receptors (PRRs), resulting in activation of transcription factors interferon regulatory factors (IRF) 3, IRF7, and nuclear factor κB (NF-κB) production of pro-inflammatory cytokines, chemokines, and interferons. RSV and IAV have also been shown to increase expression of cell surface TLR4 and TLR10 PRRs that lead to NF-κB induced transcription of pro-inflammatory cytokine genes. Both viruses are detected by NLRP3 NLRs and activate the inflammasome pathway and downstream expression of IRF3 and NF-κB dependent immune responses, as well as direct production of interleukin (IL)-1B and IL-18.

In this review, we discuss influenza- and RSV-induced immune factors that contribute to inflammation, and the recruitment of various leukocyte populations to the lung parenchyma and airway space. The recruitment of immune cells to the sites of infection is dependent on virus sensing and the release of inflammatory mediators. Influenza virus and RSV are both single-stranded RNA viruses that modulate the host innate immune response through similar pathways. Once these viruses gain access to the respiratory tract, the innate immune response is initiated by the pattern recognition receptors (PRR) of the lung microenvironment that are capable of recognizing pathogen-associated molecular patterns (PAMPS) of the invading virus [143]. RSV and influenza virus infections can activate PRRs that include Toll-like receptors (TLR), retinoic acid-induced gene I like receptor (RIG-I; RLR) and nucleotide-binding domain (NOD)-like receptors (NLR) (Figure 1) [132,143,144,145,146]. PAMP interaction with PRRs induces innate cytokines and chemokines that drive and promote inflammation and the recruitment of immune cells to the sites of infection in the upper airway and lungs. These PRRs are able to recognize multiple forms of RNA and respond by initiating intracellular signaling pathways and nuclear trafficking of transcription factors involving nuclear factor-κB (NF-κB), interferon regulatory factors (IRFs) and activating transcription factor 2 (ATF-2)/c-Jun [143,144,147].

### 4.1. Toll-Like Receptors

Toll-like receptors are distributed into two general groups: (1) those that recognize proteins and expressed on the plasma membrane (TLR1, TLR2, TLR4, TLR5 and TLR6) including lipopolysaccharides (LPS), flagellin and other proteins, and (2) those that recognize nucleic acids presented intracellularly within the endosomal compartment (TLR3, TLR7, TLR8 and TLR9) [148,149,150]. TLRs are expressed on many immune cells types including dendritic cells, macrophages, B and T cells, and neutrophils, as well as non-immune cells, such as fibroblast cells, epithelial cells, and keratinocytes [148,151]. Virus detection by TLRs activates downstream transcription factors that regulate cytokine and chemokine expression, including NF-κB, IRFs and ATF-2/cJun [143,144,147]. For the influenza virus and RSV, the TLR3 and TLR7/8 responses are involved in the detection of dsRNA and ssRNA, respectively [148]. The TLR3 is the most abundant and constitutively expressed receptor in human respiratory epithelial cells [152]. During infection, the TLR3 and TLR7/8 PRRs detect viral nucleic acids generated during IAV and RSV replication, triggering the production of pro-inflammatory cytokines and chemokines and inducing an antiviral response, while also negatively contributing to the pulmonary immunopathology. Other TLRs (TLR4 and TLR10) have been shown to have a role in the innate immune responses to IAV and RSV infections with an increase in TLR expression upon infection, ultimately resulting in an increase of pro-inflammatory cytokine production that sensitizes lung epithelium to other PAMPs (i.e., LPS and endotoxin) [153,154,155].

### 4.2. RIG-I-Like Receptors

Retinoic acid-induced gene I like receptor (RIG-I) and MDA5 are cytoplasmic PRRs critical for sensing both viral dsRNA and ssRNA in most cells, including alveolar macrophages, conventional dendritic cells, and lung epithelial cells [156]. The RLRs are activated by viral nucleic acid containing a 5′ triphosphate moiety and forming secondary “panhandle” double stranded RNA structures, which aide in the ability of the RIG-I to distinguish viral RNA from self [157,158]. Detection of either RSV or IAV viral RNA by the RLR cytoplasmic sensors results in recruitment and activation of the mitochondrial antiviral signaling protein (MAVS), leading to induction of the NF-κB and IRF3 pathways and production of pro-inflammatory cytokines and type I and II interferons (IFNs) [158,159]. 

### 4.3. NOD-Like Receptors 

The multi-protein inflammasome complexes, comprised of NLRs, the adapter (apoptosis-associated speck-like) ASC protein and pro-caspase-1 are activated upon cleavage of pro-caspase-1 into its active form [160,161]. Once in the active form, the complex proceeds to cleave the pro-IL-1β and pro-IL-18 into IL-1β and IL-18, which in turn mediate the inflammatory response to RSV and IAV [161,162,163,164]. The NLR pyrin domain containing 3 (NLRP3) is the most characterized NLR in the context of virus infections, where activation results in initiation of the inflammasome pathway and downstream activation of the NF-κB and IRF3 induced pro-inflammatory response. NLRP3 is expressed by a variety of myeloid cells that include monocytes, dendritic cells, neutrophils macrophages and respiratory epithelial cells [161]. NLRP3 is able to sense both RSV and IAV infections resulting in increased NLRP3 expression in the lungs, as well as immune cells recruited to the site of infection [161,165]. Viral proteins are able to induce the inflammasome cascade (the short hydrophobic (SH) protein of RSV, as well as the M2 and PB1-F2 of influenza) and while activation of the NLRP3 is important for virus clearance, a consequence of activation is an exacerbated inflammatory response in severe cases of IAV [161,164,165,166]. Another NLR shown to be influenced by RSV and IAV is NOD2, where both viruses result in increased NOD2 expression and subsequent activation of both IRF3 and NF-κB [167,168].

### 4.4. RSV and Influenza Induced Immune Mediators

Interferons, cytokines, chemokines are mediators of the inflammatory immune response of the respiratory tract [143]. These hormones facilitate immune and non-immune cell communication and drive leukocyte trafficking, epithelium activation, cellular proliferation and differentiation, and initiation of the adaptive immune response [143,169]. Not only do cytokines activate, regulate and control the immune response, they can establish an antiviral state, and facilitate clearance of virus, and they have been shown to promote destructive immunopathology during infection [143,170].

A role of PRRs is to initiate the activation and nuclear translocation of two transcription factors, IRF3 and NF-κB, that induce the production of pro-inflammatory cytokines and chemokines that coordinate the inflammation response and the recruitment of immune cells to the sites of infections. These immune cells include neutrophils, eosinophils, macrophages, monocytes, dendritic cells, memory T cells, Th1 cells and natural killer (NK) cells [146,162]. The influx of recruited immune cells then stimulate the secretion of a second round of cytokines, which ultimately can cause increased pathology associated with inflammation. NF-κB activation by IAV and RSV induces the expression of peculiar pro-inflammatory cytokines and chemokines. For example, RSV infection initiates the production of IFNα, IFNβ, monocyte chemoattractant protein 1(MCP1), IP10/CXCL_10_, RANTES/CCL_5_, IL6 and IL8/CXCL_8_, while IAV triggers secretion of IFNα, IFNβ, IFNγ, RANTES/CCL_5_, IL8/CXCL_8_, IL6, IL-12p40/p70 and granulocyte colony-stimulating factor (G-CSF)[171,172,173,174,175]. An exacerbated increase of pro-inflammatory cytokines and influx of immune cells to infected tissues is directly linked to disease severity and poor prognosis [135,176,177]. A portion of RSV-infected children that develop severe disease, such as bronchiolitis and pneumonia, have been linked to recurrent childhood wheezing and higher rates of asthma than children that had uncomplicated RSV infections, especially within the first 6 months of life [137,138,142,178]. Like RSV, IAV may cause ARDS [134,136]. Patients that develop ARDS and died from IAV were shown to have hypercytokinemia and massive pulmonary pathology, where histopathology consisted of inflammation, necrosis of the large airway epithelium, alveolar edema, hemorrhage and diffuse alveolar damage [136,179]. Several studies have examined cytokine profiles from patients that developed fatal ARDS as compared to those that presented mild disease. Collectively, the cytokines elevated in severe cases of 2009 H1N1 infection have been shown to have exacerbated increases of most pro-inflammatory cytokines included G-CSF, tumor necrosis factor (TNFα), IFNα2, IL-1α, IL-1b, IL-2, IL-6, IL-8,IL-9, IL-10, IL-12, IL-15, IL-17, IL-23, IP-10, MCP1 and macrophage inflammatory protein 1 β (MIP1β) [133,134,180,181]. 

## 5. Immune Evasion

Viruses employ countermeasures to attenuate the host response to infection and replication. Classically, nonstructural (NS) proteins have been shown to mediate a role in immune evasion, and facilitate their effects through interactions with cellular factors known to regulate the immune response to infections [182]. The influenza virus NS1 protein inhibits RIG-I/interferon-beta promoter stimulator 1(IPS-1) complexes and blocks type I IFN expression and pro-inflammatory cytokine expression [183,184,185]. NS1 proteins expressed by some influenza virus strains have been shown to inhibit the maturation of cellular mRNA and subsequent nuclear export by binding and blocking the host’s cellular cleavage and polyadenylation specificity factor (CPSF), thereby potentially attenuating cytokine production and export by infected cells [186].

It is important to note that RSV encodes not only NS proteins but also additional proteins to modulate the host’s immune response. The nonstructural proteins 1 and 2 (NS1 and NS2) work antagonistically to inhibit the IRF3 mediated transcription of type I and II IFNs, i.e., IFNα, IFNβ and IFNγ, in RSV-infected epithelial cells [187]. The NS proteins induce increased levels of suppressors of cytokine signaling (SOCS) proteins-1 and -3, which work through a negative feedback loop involving the Janus kinase—Signal Transducer and Activator of Transcription (JAK-STAT) pathway to regulate the activation and expression of IFN genes [188,189]. The RSV G protein also exhibits several immunomodulatory features that help control the immune response to infection, such as cytokine mimicry, inhibition of TLR activation pathways, and secretion of soluble forms to bind and disable neutralizing antibodies [190,191,192,193]. Interestingly, the RSV G protein contains a CX3C (fractalkine) chemokine motif capable of binding to the fractalkine CX3CR1 receptor to modify leukocyte trafficking and block RSV-mediated pathogenesis [191,194,195]. In addition, the RSV G protein is also able to attenuate type I IFN and interferon-stimulating gene (ISG)-15 expression through SOCS-1 and -3 induction, through interference of TLR signaling [190].

## 6. Therapeutic Potential of Targeting CRM1

Despite substantial research efforts to develop and implement antiviral measures against RSV and influenza virus, there has only been modest progress. While annual influenza vaccines are available, antigenic variations and poor strain matching ultimately reduce the efficacy of the vaccine [65]. For RSV, there is no available vaccine, and a handful of vaccine candidates are being clinically tested. Both viruses have US FDA approved antiviral drugs as discussed earlier, but these have limited effectiveness, requiring administration shortly following infection to have therapeutic value. In addition, overuse of antivirals has put selective pressure on the virus to develop mutations, driving the outgrowth of resistant strains. There is a significant need for novel antivirals and the focus of current research has shifted from the virus to targets within cellular pathways exploited by the virus for replication. The challenge is to develop an antiviral drug that is well-tolerated with minimal cytotoxicity and effective against a wide range of viruses. In this review, we explore the anti-influenza virus and anti-RSV capability of selective inhibitors of nuclear export (SINE) that specifically targets the CRM1 nuclear-cytoplasmic export pathway. Since RSV and the influenza viruses utilize the nucleus during part of their viral lifecycle, targeting the nuclear export machinery is a good antiviral approach that could act not only by inhibiting viral replication but also by attenuating the immune response by interfering with the regulation of transcription factors and production of pro-inflammatory cytokines and chemokines. There are several reviews covering CRM1 structure, function and applications targeting CRM1 as a cancer chemotherapeutic [196,197,198,199]. This review will provide an overview of CRM1 and the rationale for targeting nuclear export as a feasible antiviral strategy against influenza and RSV infections.

### 6.1. CRM1 and Nuclear Export

Nuclear import and export of all large proteins (>40 kDa) are mediated by transporter molecules and members of the karyopherin-β family of proteins. There are currently19 members of the karyopherin-β family, where each molecule is responsible for the transport of cargo proteins and RNA [196,200]. Nucleocytoplasmic transport occurs via the highly selective nuclear pore complex (NPC). The NPC is one of the largest multimeric protein complexes in the cell at 125 MDa, comprised of nearly 100 different nucleoporins [201,202,203]. While there are seven identified karyopherin-β export proteins, CRM1 is the best characterized of the nuclear export proteins and is conserved among fungi, yeast, mice, and humans [196,204]. It is a ubiquitous nuclear transporter protein that mediates the nuclear to cytoplasmic export of over 240 large proteins and RNA moieties that encode hydrophobic NES motifs [201,202,205,206,207,208,209]. Hydrophobic NES-bearing cargos bind within the Cys528 active site of the hydrophobic NES binding grove located on the outer convex surface of CRM1, which adopts three possible confirmations based on its ability to bind NES-bearing cargo and RAs-related nuclear guanosine triphosphate (RanGTP): (1) inactive with a closed NES groove unable to bind cargo; (2) active with open NES grove bound to RanGTP and cargo; and (3) intermediate with cargo bound in the absence of RanGTP [198]. Export is initiated in the nucleus where it binds the cargo and RanGTP to form a trimer that actively transports the cargo across the NPC via facilitated diffusion. Once the complex is transported to the cytoplasm, the RanGTP is hydrolyzed into RanGDP, resulting in the dissociation of the trimer and release of the cargo into the cytoplasm [208,209,210]. The CRM1 and RanGDP are then recycled back to the nucleus where they can undergo another round of export. The energy required to facilitate the CRM1 export process is generated by a high RanGTP concentration gradient in the nucleus, driving the facilitated nuclear export of cargo proteins to the cytoplasm [210].

### 6.2. SINE Inhibition of Influenza and RSV

Selective inhibitors of nuclear export (SINE) compounds are designed to bind exportin receptors and inhibit the cytoplasmic transport of NES-bearing cargo proteins. Preliminary studies have focused on the use of SINE compounds as anti-cancer therapeutics, since the inhibition of CRM1-mediated export results in nuclear retention of tumor suppressing proteins (TSP) and cell cycle regulators (CCR), allowing them to impose cell cycle control and selective apoptosis in several tumor types including ovarian, pancreatic cervical, mammary, and lymphoma cancer cells [211,212,213,214]. In recent studies, SINE compounds have shown antiviral activity against viruses that utilize the nucleus as part of their viral life cycle. While LMB is the prototypical inhibitor used to characterize CMR1 trafficking, its irreversible binding, and off-target effects cause it to be highly toxic and not a suitable antiviral candidate [215,216]. Karyopharm Therapeutics has recently developed first-in-class, novel selective inhibitors of nuclear export (KPT-SINE) using molecular modeling to screen and identify compounds that interact with the NES groove of CRM1 [208,209,217]. KPT-SINE compounds are reversible inhibitors of CRM1-mediated nuclear export, and the crystal structure revealed that a SINE molecule binds within the NES binding pocket of CRM1 and appears to penetrate deeper into the groove than LMB [217]. One of the KPT-SINE compounds, Verdinexor (KPT-335), is an orally bioavailable well-tolerated compound that has exhibited efficacy as an anti-cancer and antiviral drug. In initial preclinical work, KPT-335 was shown to effective cancer chemotherapy against canine lymphomas, but our group has also demonstrated the potential of KPT-335 as a novel broad-spectrum antiviral against various IAV and IBV strains and RSV (unpublished) [14,211,213,218,219].

**Figure 2:**
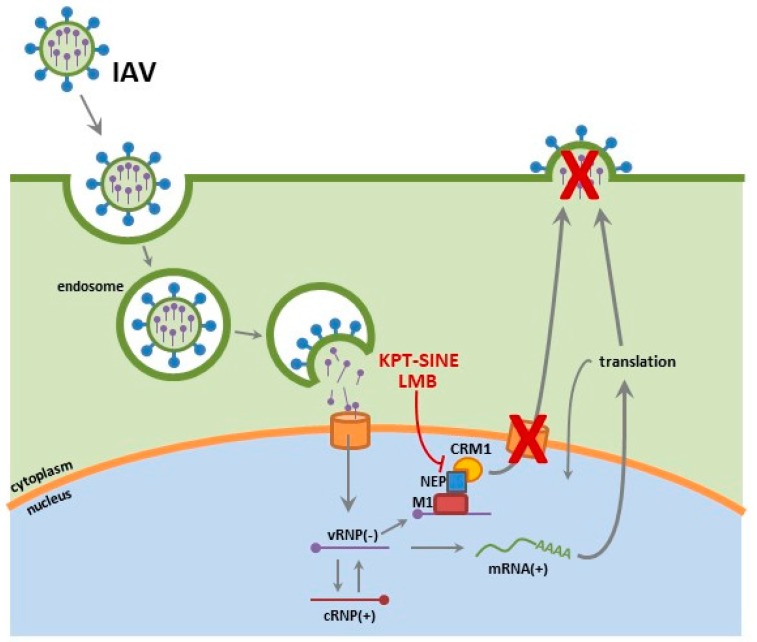
Select inhibitors of nuclear export (SINE) interfere with CRM1 mediated export of the IAV viral ribonucleoproteins (vRNPs), thereby sequestering them to the nucleus and inhibiting late stage assembly processes.

We examined the potential of KPT-335 as a broad-spectrum antiviral against various influenza strains in vitro and in vivo [14,218]. KPT-335 was shown to be efficacious at reducing virus replication of pandemic H1N1 (A/California/04/09), highly pathogenic H5N1 avian influenza strain and the recently emerged H7N9 in vitro [14]. Furthermore, KPT-335 treatment was also shown to reduce virus burden, lung pathology and cytokine expression in mice and ferrets against pandemic H1N1 (A/California/04/09) and H3N2 (A/Philippines/2/82) [218]. We assessed the expression of key NF-κB dependent cytokines and demonstrated substantial reduction in the expression of IFNγ (*p* ≤ 0.05), TNFα (*p* ≤ 0.01), IL-1β (*p* ≤ 0.01), and IL-6 (*p* ≤ 0.05). In our most recent report, KPT-335 treated mice had considerably lower IFNγ and TNFα (*p* ≤ 0.05) in bronchoalveolar lavage fluid (BALF) and reduced levels of IL-6 and IL-12p40 at day 2 through day 4 pi. In addition, there was no substantial increase in inflammatory cell infiltrates (macrophages, natural killer cells, T cells and granulocytes) detected in the mice. While in ferrets, lower numbers of infiltrating leukocytes were observed in all KPT-335 treated ferrets as early as day 2 post-infection (pi) and turned to significant reductions by day 4 post infection. Ferrets that received KPT-335 up to 25 mg/kg once daily or 10 mg/kg twice daily showed reduced expression of IL-12p40 (*p* ≤ 0.01), IFNγ and TNFα (*p* ≤ 0.01) in nasal exudates as early as day 2 pi. We demonstrated that KPT-335 mediates its effect by disrupting CRM1-NEP binding, resulting in potent and selective nuclear retention of the vRNPs and thereby blocking their ability facilitate viral replication and assembly processes (Figure 2).

**Figure 3:**
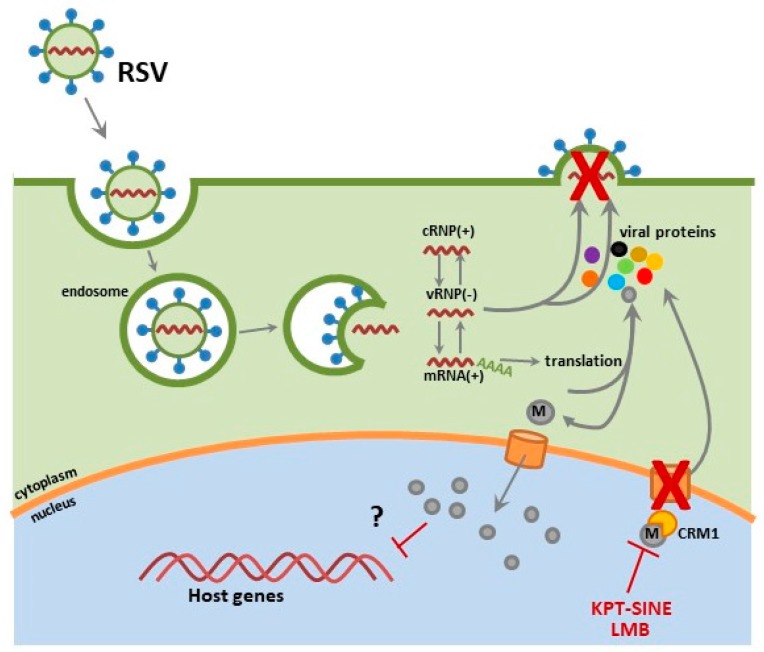
Respiratory syncytial virus (RSV) matrix (M) protein translocates to the nucleus early in the infection life cycle and inhibition of CRM1 export by leptomycin (LMB) or KPT-SINE reduces viral production and results in M protein nuclear accumulation.

We have preliminary data demonstrating KPT-335 as a safe and effective anti-RSV therapeutic. In these studies, therapeutic administration (2 hours pi) of KPT-335 reduces replication of RSV A2, RSV Long and RSV B1 strains. Slight RSV M nuclear accumulation was detected in KPT-335-treated respiratory epithelial cells; thus, we hypothesize that the observed virus reduction was due to nuclear sequestering of the RSV M protein, where it is unable to facilitate viral assembly and budding processes essential for virus replication (Figure 3). In addition, the expression of IL-8, RANTES, and growth regulated protein alpha (GROα) and MCP-1 was reduced in KPT-335 treated A549 cells (i.e., respiratory epithelial cells) as compared to the mock-treated controls, although not statistically significant. We are currently investigating the in vivo efficacy of KPT-335 against RSV in an RSV-experienced mouse model. We propose that KPT-335 treatment will reduce viral burden, lung pathology and cytokine expression similar to what was observed in the influenza virus studies. We hope to demonstrate the broad-spectrum antiviral activity of KPT-335 against RSV A and B strains in a RSV-experienced mouse model. These results will complement previous studies showing KPT-335 efficacy against strains of IAV. Additionally, we seek to better understand the interactions of the RSV M protein with host cellular factors and determine their influence on cellular processes.

### 6.3. CRM1 Inhibition of NF-κB

Inhibition of nuclear export works not only to block export of viral proteins but also interferes with downstream immunomodulatory responses triggered by early stages of the viral infection cycle. It is the recognition of viral PAMPs present at each stage of pathogenesis (i.e., entry, fusion, transcription, translation and assembly) that drives activation of cellular and downstream immune responses. SINE compounds work to block nuclear to cytoplasmic export of viral and immune mediators that utilize CRM1 as part of their regulator pathways, resulting in attenuated functionality.

**Figure 4:**
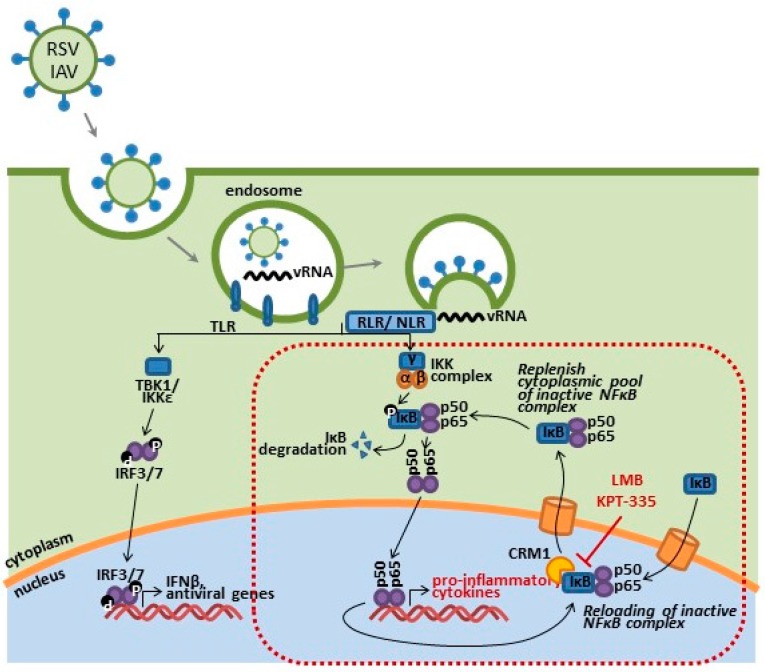
Respiratory syncytial virus (RSV) and influenza A virus (IAV) ssRNA and dsRNA moieties are detected by PPRs, triggering the downstream production of pro-inflammatory and interferon responses. KPT-SINE compounds may also act as immunomodulatory drugs disrupting the activation and regulation of IκB (inhibitor of NF-κB), as well as other transcriptional factors initiated during viral infection.

Select inhibitors of nuclear export (SINE) compounds, while effective as a possible antiviral drugs and chemotherapeutics, have exhibited anti-inflammatory activities by disrupting NF-κB regulation and activation pathways. The NF-κb inhibitor, IκB, utilizes CRM1 to repress NF-κB signaling within both the nuclear compartment and the cytoplasm [220,221]. The IκB bound to cytoplasmic NF-κB renders it inactive and thereby prevents and trafficking of NF-κB subunits (RelA and p50) to the nucleus; additionally, IκB also represses postinduction NF-κB activation through interference on NF-κB/DNA binding and subsequent expression of cytokine genes. This is a tightly regulated pathway, and efficient nuclear export of IκB is required for the replenishment of cytoplasmic pools of NF-κB/IκB complexes and subsequent regulation of NF-κB activation. The inhibition of CRM1 export results in nuclear retention IκB and NF-κB subunits, thus leading to inactivation of NF-κB signaling [220,222,223,224]. We have observed nuclear accumulation of NF-κB subunits following KPT-335 treatment in RSV and IAV infected cells, as compared to a mock-treated control. NF-κB activation has been shown to correlate with disease severity in influenza and RSV infections, along with countless other infectious pathogens, through the upregulation of pro-inflammatory cytokines and infiltration of immune cell to sites of infections (Figure 4) [146,147,148,149,156].

## 7. Conclusions

The influenza virus and RSV are a major public health concern. It is clear that a different approach needs to be considered to combat outbreaks and reduce the burden of these respiratory pathogens. SINE compounds are well tolerated, orally bioavailable drugs that may provide solutions to the outstanding issues with currently available antivirals, such as drug resistance, poor tolerability and reduced efficacy. SINE targeting of CRM1 could have a dual therapeutic effect: (1) repressing of the NF-κB dependent cytokine response and elimination of virus-associated immunopathology; and (2) reduction in the nuclear export of critical viral protein(s), thereby attenuating late stage virus replication processes. Our preliminary studies have provided the initial framework for future human clinical evaluations of KPT-335 as an antiviral against RSV and influenza virus. Given that many viruses utilize the nucleus as part of their viral life cycle, KPT-335 inhibition of CRM1-mediated export could be effective against a number other viruses, including human immunodeficiency virus (HIV), Hepatitis B, Hepatitis C, and Herpes simplex viruses [182]. The repurposing of SINE compounds from cancer chemotherapeutics to safe and effective antivirals and anti-inflammatory drugs increases the therapeutic potential of these compounds.

## Figures and Tables

**Table 1:** Therapeutics under investigation against respiratory syncytial virus and influenza viruses.

Drug Name	Target	Reference(s)
**RSV inhibitors**		
GS-5806	RSV F protein	[102,103,104]
NMSO3	RSV G protein	[105,106]
RSV 604 (benzodiazepine)	RSV N protein	[107,108]
VP-14637	RSV F Protein	[109,110,111]
AZ-27	RSV L protein	[112]
ALS-008176	RSV L protein	[113,114]
Nitazoxanide	Pyruvate: ferredoxin oxidoreductase (PFOR) enzyme dependent electron transfer reaction	[115]
**IAV inhibitors**		
Nitazoxanide	HA protein	[115,116]
Favipiravir	RNA dependent RNA polymerase complex	[117,118]
VX-787	PB2 protein	[119,120,121]
AL-794	PA protein	[122]
Nucleozin	NP protein	[123,124]
DAS181	Fusion inhibitor	[125,126,127]
**Immunomodulatory drugs**		
Eritoran (E5564)	TLR4 antagonist	[128,129]
Rosiglitazone/pioglitazone	Peroxisome proliferator-activated receptor γ antagonist	[130]
Azithromycin (macrolides)	Interferon-augmenting	[131]

Abbreviations: F: Fusion; G: Attachment; N: Nucleoprotein; L: Large Polymerase; HA: Hemagglutinin; PB2; RNA polymerase Basic 2; PA: Polymerase Acidic; NP: Nucleoprotein; TLR4: Toll-like Receptor 4.
